# Urban population prediction based on multi-objective lioness optimization algorithm and system dynamics model

**DOI:** 10.1038/s41598-023-39053-1

**Published:** 2023-07-22

**Authors:** Dong Li, Yanyan Yu, Bo Wang

**Affiliations:** 1 School of Economics and Management, Xi’an University of Posts and Telecommunications, Xi’an, 710061 China; 2 School of Modern Posts, Xi’an University of Posts and Telecommunications, Xi’an, 710061 China

**Keywords:** Computer science, Statistics

## Abstract

Population size is closely related to economic and social development and change. It is one of the primary and essential elements of overall urban development planning to formulate a population development strategy scientifically through population projections. Therefore, we propose an urban population prediction model based on a multi-objective lioness optimization algorithm and system dynamics. The multi-objective lioness optimization algorithm is used to optimize some critical parameters of the system dynamics model to reduce the subjectivity of the model construction. Taking Xi’an as an example, the validity of the model is verified, and the population size of Xi’an from 2019 to 2050 is predicted by the model. In addition, the impact of different policies and their combinations on the future population is discussed through simulations of three scenarios composed of five policy factors: birth, employment, science and technology, healthcare and education. The results show that the total population of Xi’an will peak at 147,939,242 in 2040, based on current development trends. Moreover, the five policies with the largest to smallest positive effect on population size are: employment policy, fertility policy, education policy, science and technology policy, and health policy, with employment and fertility policies having significantly larger effects than the other three. Therefore, the employment policy and the birth policy are the two most effective policies to promote population growth, and the coordinated implementation of the five policies is the fastest way to increase population size.

## Introduction

Population prediction is a significant issue that affects whether a country or a city can achieve sustainable development. It is also an essential factor for governments, enterprises, and non-governmental organizations to formulate plans^1,2^. Scientific and reasonable population projections can provide a scientific basis for the distribution of various social resources, urban development planning and the construction of public service facilities. It can also enable governments to understand potential risks in advance for risk management, helping the regular operation and virtuous cycle of society as a whole. In recent years, the demographic situation has undergone a fundamental transformation as the country’s economic and social development situation has rapidly shifted and the concept of marriage and childbearing has changed significantly. To achieve sustainable and coordinated social development, it is necessary to predict demographic development trends in a timely manner. Population policies and economic and social development plans will then be adjusted based on the results of population projections.

Considering the vital role of population in the urban development, numerous scholars have explored the demographic development trends. By combing through the above literature, it can be found that scholars have made a lot of progress in the field of population prediction, but there are also some limitations. First of all, population forecasting is a work that needs to be dynamically adjusted in time according to the latest information. With the passage of time, there is a large gap between the population status and some of the research achievements, which can no longer guide the city in the design of relevant policies. Secondly, during the research design, some studies failed to completely account for global factors or the effects of external perturbations on the regional population, resulting in low accuracy and guidance of the prediction results.

It is the specialty of system dynamics (SD) to deal with such an irregular, nonlinear, and lengthy-period complex system as population.

Although system dynamics has achieved excellent results in various fields^3–6^, it is not perfect, and there are still some problems to be solved. The most typical one is that some parameters are set randomly and inefficiently in the modeling process, which leads to deviation in prediction accuracy^7^. To solve this problem, achieve the objectivity of parameter setting and improve the efficiency of parameter setting, we propose a multi-objective lioness optimization algorithm (MOLsOA) to optimize some critical parameters in the system dynamics model. The lioness optimization algorithm (LsOA) is a single objective optimization algorithm based on population, which imitates the behaviors of team hunting and elite hunting in the hunting behavior of lionesses. LsOA was proposed by Li et al.^8^ and proved to have superior parameter optimization performance in ozone prediction. Considering that single-objective optimization algorithms fail to meet the multi-objective requirement for parameter optimization of system dynamics models, we propose MOLsOA based on LsOA, which can optimize parameters of system dynamics models for multiple objectives simultaneously. Therefore, compared with the traditional population prediction model, we believe that the combination of MOLsOA and SD model can improve the simulation capabilities of population prediction and better simulate the future trend of population.

The main contributions of this study are as follows.

1. This study examines the primary policy factors that influence urban population dynamics through the utilization of the system dynamics method. The aim is to provide reference for cities to develop scientifically informed population development plans and overarching developmental objectives. Specifically, after conducting a systematic analysis of demographic, economic, educational, medical, and other factors, a system dynamics model is constructed. The model's effectiveness is evaluated by assessing its performance. Subsequently, five policies are formulated regarding fertility, employment, science and technology, healthcare, and education. These policies are used to create different scenarios, and the quantitative analysis focuses on assessing the effects of the various policies and their combinations on population dynamics.
2. A multi-objective lioness optimization algorithm (MOLsOA) is developed by incorporating an individual location-update strategy and external elite repository into the lioness optimization algorithm (LsOA). The efficiency of MOLsOA is assessed using standard multi-modal benchmark functions (MMFs). The experimental results reveal that MOLsOA surpasses the performance of eight other multi-objective optimization algorithms for solving nonlinear multi-objective optimization problems, thereby establishing the practicality of the proposed strategy.
3. We applied MOLsOA for optimizing the parameters of the system dynamics (SD) model, which mainly includes the optimization equation parameter and the constants that are challenging to compute from available data sources. Our approach optimizes the feedback relationships amid variables in the model, mitigates the subjective selection of parameters prevalent in traditional SD modeling, and upgrades both efficiency in parameter tuning and model prediction effects. As per our knowledge, this may be the first effort utilizing a multi-objective optimization algorithm for system dynamics parameter optimization.

Other parts of this paper are arranged as follows: section “Literature review” describes the elements of population growth and related work on population forecasting methods. In section “Materials and methods”, we present a comprehensive account of our system dynamics model for population prediction and detail the process of refining the model parameters through the MOLsOA. Moreover, we evaluate the modeling output to demonstrate the reliability of our approach. In section “Empirical test”, we take Xi’an as an example to test our model. In section “Policy scenario analysis”, we design three policy scenarios and analyze the simulation results of each policy scenario in detail. Finally, section “Conclusions” summarizes the paper, discusses the main findings of this study, and explores shortcomings and future research directions.

## Literature review

### Population influencing factors

Population change is a multifaceted product influenced by various factors. From existing literature, these factors can be grouped into four main categories: natural, environmental, socio-economic, and governmental policies.

Natural factors, including fertility rates, mortality rates, population age structure, sex ratios, dependency ratios, and other demographic indicators, are crucial determinants of population growth. Numerous population prediction studies rely on natural factors. For instance, Wisniowski et al.^9^ developed a population prediction model that considers age and gender, demonstrating its flexibility and advantages using UK population data. Wei et al.^10^ conducted an empirical analysis on the factors influencing population growth, providing evidence that a balanced sex ratio can result in excessively rapid population growth. Zhong et al.^11^ demonstrated that the dependency ratio has a significant and long-term impact on the population.

Numerous studies have demonstrated the significant impact of environmental factors, such as climate, terrain, natural resources, and natural disasters, on population dynamics. For instance, Cheshire and Magrini^12^ conducted an analysis of the population in 12 metropolitan areas within the European Union, revealing that cities with more favorable weather experienced higher rates of population growth. Similarly, Lupi and Marsiglio^13^ discovered that climate change can influence birth and mortality rates. Regarding terrain and population, Feng et al.^14^ conducted an analysis and provided evidence of a strong logarithmic relationship between relief degree and population using GIS technology. Wang et al.^15^ identified that factors such as temperature and precipitation can enhance the accuracy of population prediction models. It is important to note that while the impact of natural disasters on population is not sustainable, its effects can be significant. Consequently, population forecasting should incorporate considerations of natural disasters^2^. Zhong et al.^11^ discovered that the factors influencing population growth have undergone significant changes in the context of COVID-19. Specifically, the impact of fertility support facilities and women’s education level on population has gained more importance compared to the past, while the influence of the dependency ratio has diminished.

With the growing activity of migration, its significance in predicting future populations is increasingly crucial. In population projection studies, an increasing number of scholars are considering socio-economic factors as they have a significant impact on migration.

Socio-economic factors generally encompass indicators that reflect the level of social development and can be classified into construction and economic dimensions. Firstly, the key factors reflecting the level of construction include education, science and technology, healthcare development, infrastructure construction, local public expenditure, and urbanization rates. Cheng et al.^16^, Li et al.^17^, and Zhou et al.^18^ have individually demonstrated that the development level of education, science and technology, and medical treatment significantly influence population migration. Zhu and Zeng^19^ employed econometric models to analyze the factors influencing urban population growth in 1990s China. Their findings revealed that infrastructure conditions have a significant impact on the pace of urban population growth. Rappaport^20^ and Rodríguez‐Pose and Ketterer^21^ highlight that improved infrastructure conditions facilitate attracting more people to relocate. Additionally, Wei et al.^10^ and Wang and Lee^22^ respectively identified urbanization rate and local public expenditure as crucial factors influencing population dynamics. Secondly, the economic level provides a comprehensive reflection of factors such as GDP, income, and employment, which serve as the primary internal drivers of population growth^23,24^. For instance, Etzo^25^ discovered that GDP is the decisive factor in determining immigration flows. Liu and Feng^26^ examined the factors influencing population migration using data from the 2010 6th China National Population Census. The results demonstrated that income played a crucial role in driving population migration. Wei et al.^10^ identified that the employment rate positively influences population growth and proposed that increasing the employment rate would be a long-term strategy for China to enhance population growth. Riiman et al.^27^ developed a population prediction model (ANN-LSTM) that incorporated factors such as employment opportunities, wages, real per capita income, and real average income per job. The research findings indicated that the model considering these factors yielded favorable results.

Government policy factors, such as fertility policies, migration policies, and development strategies, exert a substantial influence on population dynamics. Among these, fertility policies can induce alterations in birth rates and profoundly shape population development trends. Extensive research has been conducted by numerous scholars on populations under various fertility policies, and the findings consistently demonstrate that more lenient fertility policies are associated with higher fertility rates^28–31^. Immigration policies significantly influence the size of the floating population. Population growth rates are higher when regional development policies and social security policies are more comprehensive. For instance, Zhong^32^ conducted an analysis of the factors influencing China's urban population at different stages and concluded that population growth was influenced by factors such as the household registration system, regional development strategy, and other policy factors.

### Population prediction method

Over the years, scholars at home and abroad have studied population prediction and proposed a range of methods. The Malthusian population growth model is one of the classical models, which assumes that the population can grow indefinitely. The logistic population growth model improves on the Malthusian population growth model. That is, the maximum number of people that can be carried by natural resources is introduced into the population growth model. Yang et al.^33^ compared the Malthusian population model, logistic growth model, and linear regression analysis. The results show that linear regression has the worst performance in population prediction. Gao et al.^34^ found that the logistic model was better than the Malthusian population growth model when forecasting the long-term population. The logistic model needs to solve differential equations, which are difficult to solve, leading to large errors in prediction^35^.

As research deepened, scholars began to build population trend prediction models from birth, death, and migration perspectives. Among them, the typical method is the cohort factor method^36^, which predicts the future population change rate based on the law of the current population change rate, thereby realizing population prediction. This method performs adequately in medium-term and long-term population prediction^37^. However, the future population ratio, one of the key parameters of the cohort factor method, needs to be manually specified by researchers, and improper setting of this value will lead to a significant deviation in the prediction results^22^.

Probabilistic population prediction methods have been developed gradually in recent years. Sheng et al.^38^ found that probabilistic population prediction methods are better than cohort factor methods in population prediction. The most typical probabilistic population prediction method is the Bayesian method. Czado et al.^39^ proposed a Bayesian log-bilinear Poisson regression model to analyze the relationship between mortality and age, mortality and calendar time with the data of the male population aged 0–89 years in France from 1950 to 2000. Alkema et al.^40^ established a Bayesian model to predict the TFR of all countries by country, and the research results showed that this method has decent calibration. Wisniowski et al.^9^ proposed a totally integrated dynamic Bayesian method for population prediction according to age and gender. The results demonstrate the applicability of this approach to population forecasting.

Population forecasting is essentially a time series problem. In recent years, scholars have applied time series methods to population forecasting^30,41,42^. ARIMA^43,44^ is one of the typical time series models. The problem with ARIMA is that it has strong requirements for data and needs to meet numerous assumptions when dealing with issues^22^. These problems limit the application of ARIMA to population prediction.

The grey forecasting model^45^ is also one of the common methods of time series modeling. The advantage of the grey prediction model is that it can only use the time series characteristics of the data itself to model and predict, which is simple and adaptable and can better deal with the changes of abrupt parameters. Li et al.^46^ compared ARIMA (1, 1, 1) and grey prediction models and found that the latter had a higher fitting degree to historical data. Chen et al.^47^ found that compared with the Malthusian model, unary linear regression model, and logistic model, the grey prediction model has the highest accuracy. Recently, Liu et al.^48^ established a new grey prediction model (CFODGMW (1, 1, α)) to study the aging of China's population. Guo et al.^49^ put forward a new grey population prediction model. Comparing with other grey prediction models through actual cases, they found that the prediction accuracy of their model was higher than that of the traditional grey prediction model.

However, no matter which method is mentioned above, by their very nature it is difficult to systematically model or predict population growth by fully accounting for external environmental factors. To address this issue, several scholars have applied system dynamics to population prediction.

System dynamics has been applied in various social, economic, and environmental systems since it was proposed^50^, and now it has become one of the critical methods for studying complex systems. It is based on the causal feedback loop formed between system elements, which makes it get rid of the dilemma of pure numerical analysis and provides a foundation for its cross-application with different disciplines^7^. Currently, numerous authors have applied system dynamics models to population studies. Mielczarek et al.^51^ proposed a population system dynamics model to study population prediction and the relationship between population and medical demand. Tomaskova et al.^52^ used system dynamics to predict the total population and the number of Alzheimer’s disease cases in the European Union in 2080. In China, Wu et al.^53^ used the system dynamics model to predict the future population changes in China after the implementation of the two-child policy, and they suggested that China's fertility policy still needs to be more relaxed. Recently, Li et al.^54^ combined system dynamics with the push–pull theory of population migration and applied it to the study of population migration. It is shown that the combination of the system dynamics and the push–pull theory can better model the population migration process.

### Research gaps

After conducting a comprehensive analysis of existing literature, it is clear that researchers have primarily focused on population forecasting of national-level populations^10,39,48^ while comparatively neglecting the subject of urban population projection^18^. Notably, although cities and nations share some similarities in terms of projected population outcomes, variations exist as well. First, population forecasts at both national and urban levels commonly incorporate indicators such as birth rate, mortality rate, gross domestic product (GDP), consumption level, and migration rate. However, these factors exhibit substantial variations in their influence on national and urban population projections. For instance, migration rates have distinct effects on the prediction of national and urban populations. Immigrants face higher economic costs, more significant differences in cultural customs, and greater obstacles due to different national political positions when moving from one country to another compared to migration between cities. Consequently, mobility between countries is typically lower than migration between cities. Therefore, mobility has a more substantial impact on population projections at the city level than on the national level. Second, urban population predictions require the consideration of additional specific factors, including economic development status, population status, employment opportunities, and infrastructure construction. These factors are crucial for modeling and studying cities based on their historical trends, current development status, and development goals. However, accurately incorporating these factors into national population forecasting models presents challenges. Lastly, conclusions drawn from research objectives at the national level may not effectively inform cities in devising appropriate policy designs, given the variations between cities and their distinct future development goals. Consequently, focusing our research efforts on cities can offer practical guidance and support for urban development, while simultaneously enhancing research outcomes in the domain of urban population prediction and drawing scholarly attention to urban population issues.

Due to the advantages of system dynamics in the study of complex systems, many scholars have applied this method to study population systems with favorable results. Regrettably, the issue of subjective parameter settings in system dynamics has yet to be adequately addressed. As far as our knowledge extends, the only study to optimize variables in system dynamics models using genetic algorithms has been put forth by Yu and Wei^55^. They considered certain variables in the model as time-dependent functions and constructed variational equations with time as an independent variable. In, time and its main dependent variables are fully taken into account in equations with only one variable. Subsequently, a genetic algorithm was employed to optimize the coefficients in the variational equation by utilizing the inverse of the error between the simulation and the actual data as the sole optimization objective. While this approach enhances the simulation accuracy of the model, it does not fully consider the interrelationships between the different variables.

Compared to this study, our research focuses on enhancing the feedback relationship among internal variables within the system dynamics model. Specifically, our objective is to optimize the coefficients in the equations between different variables. Additionally, we also aim to optimize the constants in the model that cannot be precisely computed from real data. This optimization process aims to further mitigate the subjectivity of parameter settings and improve the predictive performance of the model. Moreover, we propose an innovative approach by employing multi-objective optimization algorithms for parameter optimization. Unlike single-objective algorithms, multi-objective algorithms enable the simultaneous consideration of multiple objectives and trade-offs among potentially conflicting objectives. As a result, they provide more efficient, effective, and comprehensive solutions to complex problems.

Additionally, during collating historical documents, we find that many scholars have explored how economic, environmental, and public services affect population trends while examining demographic issues. However, these discussions have been primarily qualitative in nature and lack quantitative analyses. Hence, this study systematically analyzes demographic influences, constructs a system dynamics model, and quantitatively evaluates the effects of various policies and their combinations on future population development.

## Materials and methods

### Causal loop and flow diagrams

This study aims to predict the future trends of the population under different policies. Considering the integrality and global nature of the urban population forecasting system, the feedback loop of each factor affecting population development and change is taken as the system boundary of the dynamic model of the urban population forecasting system. Previous studies have found that factors affecting population development mainly include the economy^25^, education^56^, and medical treatment^57^. Therefore, urban population forecasting system is divided into four sub-systems: population, economy, education and healthcare. There are complex causal relations among the different subsystems.

Numerous studies have shown that economic, educational and medical development can improve the living environment of cities and enhance their attractiveness to foreign populations, thus increasing the overall population of cities in terms of population migration. However, continued population growth will lead to the consumption of resources and deterioration of social living conditions, which will not be conducive to the development of the economy, education and healthcare. As a result, the economy, education and healthcare form a negative feedback loop with the population, and their interactions constitute a complex dynamic process.

**Figure 1:**
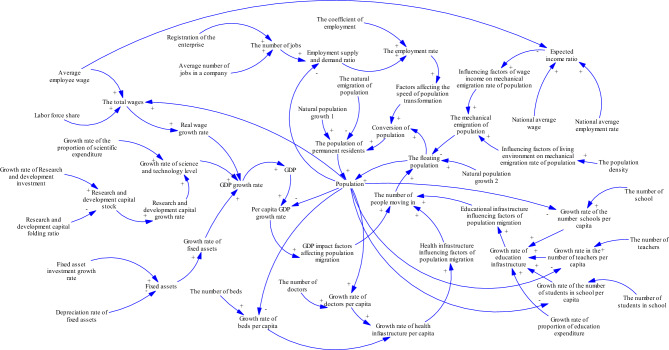
Causal loop diagram of the urban population forecasting system.

The causal loop diagram of the system is shown in Fig. 1, which includes the following seven main feedback loops:

1. Population➜ +The number of people moving in➜ +The floating population (➜+ Conversion of population➜ +The population of permanent residents)➜ +Population.

Higher population in a city results in an influx of floating population, which further contributes to its increased population density. Therefore, this is a positive feedback loop.

2. Population➜ +The population density➜ +The influencing factors of living environment on mechanical emigration rate of population➜ +The mechanical emigration of population➜−Population.

When population growth results in local population density becoming saturated, it triggers the influence factors of living environment that, in turn, motivate residents to emigrate elsewhere. This migration contributes to a drop in population, resulting in a negative feedback loop.

3. Population➜ +The total wages➜ +The real wage growth rate➜ +The GDP growth rate➜ + GDP➜ +The per capita GDP growth rate➜ +The GDP impact factors affecting population migration➜ +The number of people moving in➜ + Population.

As the population grows, so does the labor force, ultimately having a favorable impact on the total wages. A crucial constituent of GDP, higher total wages equate with increased GDP. With increasing levels of economic development (measured by the economic impact factor), more individuals relocate to cities, resulting in further population expansion. This creates a positive feedback loop.

4. Population➜− Per capita GDP➜ +The per capita GDP growth rate ➜ +The GDP impact factors affecting population migration➜ +The number of people moving in➜ +Population.

Loop (3) illustrates the positive impact of population growth on GDP. But exceeding GDP growth with population expansion leads to an adverse effect on per capita GDP, weakening economic impact factors, reducing in-migration, and decreasing the overall population size. For these reasons, this feedback loop operates negatively.

5. Population➜−Employment supply and demand ratio➜ +The employment rate➜ +The factors affecting the speed of population transformation➜ +Conversion of population ➜ +The population of permanent residents/-The floating population➜ +Population.

When the population growth rate exceeds the employment growth rate, it results in a smaller employment supply–demand ratio, leading to a reduction in the employment rate. Furthermore, the factors influencing the rate of population transformation affected by the employment rate also decrease, thereby reducing the rate of transition from the floating population to permanent residents. Floating populations are more prone to emigration probabilities relative to permanent populations. Consequently, declining ratios of employment-supply leads to heightened rates of emigration, which results in a reduction of the population. Hence, producing a negative feedback loop in the system.

6. Population➜−The growth rate in the number of teachers per capita/The growth rate of the number schools per capita/The growth rate of the number of students in school per capita➜ +The growth rate of education infrastructure➜ +The educational infrastructure influencing factors of population migration➜ +The number of people moving in➜ + Population.

Teachers, the number of schools, and students are key variables that constitute the educational infrastructure. When their growth rate is lower than that of the population, the increase in teachers per capita, schools per capita, and students per capita exhibits a declining trend, resulting in a decrease in the growth rate of educational infrastructure. Additionally, the educational infrastructure impact factor, which is influenced by the growth rate of educational infrastructure, also diminishes. Consequently, the influx of people decreases, leading to a decline in the population. Thus, this represents a negative feedback loop.

7. Population➜−The growth rate of doctors per capita/The growth rate of beds per capita ➜+The growth rate of health infrastructure per capita➜+The health infrastructure influencing factors of population migration➜ +The number of people moving in➜+Population.

Similar to loop (6), when the growth rate of the number of doctors and beds lags behind that of the population, the per capita growth rate of medical infrastructure exhibits a declining trend. As a result, the impact factors of medical infrastructure composed of these variables also diminish, leading to a decrease in the population influx. Subsequently, the overall population decreases. Therefore, this system loop also represents negative feedback.

**Figure 2:**
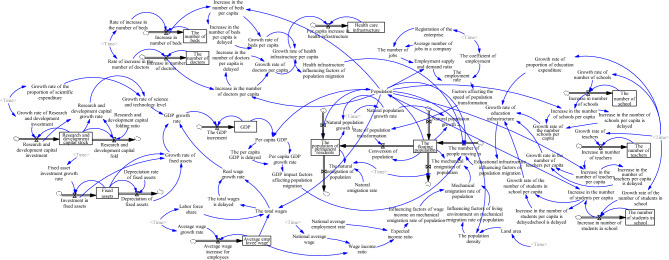
Stock flow diagram of the urban population forecasting system.

Based on the causal loop diagram analysis mentioned above, a dynamic model of the urban population system is established (see Fig. 2).

The leading indicators for each subsystem are as follows.

Population subsystem: the population of permanent residents, the floating population, natural population growth rate, natural emigration rate, mechanical emigration rate of population, factors affecting the speed of population transformation, influencing factors of wage income on mechanical emigration rate of population, and influencing factors of living environment on mechanical emigration rate of population, etc.

Economy Subsystem: GDP, fixed assets, average employee wage, research and development capital stock, fixed asset investment growth rate, depreciation rate of fixed assets, average wage growth rate, the growth rate of research and development investment, research and development (R&D) capital folding ratio, the growth rate of the proportion of scientific expenditure, GDP growth rate, and GDP impact factors affecting population migration, etc.

Education subsystem: the number of schools, the number of teachers, the number of students in school, the growth rate of the number of schools, the growth rate of teachers, the growth rate of the number of students in school, the growth rate of the proportion of education expenditure, and educational infrastructure influencing factors of population migration, etc.

Medical subsystem: health care infrastructure, the number of beds, the number of doctors, the rate of increase in the number of beds, the rate of increase in the number of doctors, and health infrastructure influencing factors of population migration, etc.

### Optimization of the SD model parameters

#### Design the SD model equations

Relations between the variables in the system are found from historical data and the equations of the variables are constructed from these relations, which is the basis of the building system dynamics model. The parameters in the variable equations need to be determined in light of the actual situation of the city. However, in the existing system dynamics research, the parameter settings are often based on the subjective judgment of the researcher, which can lead to significant deviations in the prediction results. To solve this problem, an optimization algorithm is introduced to optimize the parameters automatically to improve the reliability and objectivity of the parameters and improve the accuracy of the population prediction.

By combing through the dynamical model of the population prediction system, and depending on the availability of data, the natural population migration rate and the employment coefficient in the constant are used as optimization parameters. At the same time, the interaction of the various variables of the system is analyzed, the equations of the system dynamics are established, and coefficients of no practical significance are added to the equations for correction, with reference to the existing results. There are altogether 19 such coefficients. In summary, we identified a total of 21 parameters to be optimized, including 2 constants and 19 coefficients. The variable equations of parameters to be optimized are shown in Eqs. (1)–(11), where $x_{i}$ are the parameters to be optimized. To make the formula more concise, specific abbreviations are used for the variables. The table of comparison letters is given in Table 1, and the equations for each variable are described as follows.

1. Population immigration. Reviewing the available studies, it can be seen that the economic, medical and educational influences on the regional population are positive. Therefore, all three factors are included in the selection of influencing factors of the number of people moving in (NI) (Eq. 1). Specifically, the economic impact factors affecting population migration (GDPF) reflect the Gross Domestic Product (GDP), based on the way GDP is calculated in economics, growth rate of fixed assets (FR), real wage growth rate (RSR) and growth rate of science and technology level (SLR) are taken as specific indicators of economic impact factors (Eq. 2). Among them, the growth rate of science and technology level is composed of two hands (Eq. 3): R&D capital growth rate (RDP) and growth rate of the proportion of scientific expenditure (STR), which can represent the level of regional science and technology development. Health infrastructure influencing factors of population migration (MF) takes the growth rate of doctors per capita (ADA) and growth rate of beds per capita (ABA) as specific indicators (Eq. 4). Educational infrastructure influencing factors of population migration (ERF) takes growth rate of the proportion of education expenditure (ER), the growth rate of teaching facilities (ENP), growth rate of the number of students in school per capita (SRP) and growth rate in the number of teachers per capita (TRP) as specific indicators (Eq. 5).
2. Population emigration. Equations (6–9) describe the relationship between variables affecting urban population emigration. Natural emigration rate (NO) refers to the ratio of emigration of the population to the total population caused by changes in residents’ conditions (Eq. 6). The opposite is the mechanical emigration rate of population (MO), which refers to the ratio of the emigration of the population to the total population caused by the deterioration of the regional living environment and the decrease of wage income (Eq. 7). Studies have found that the lower the per capita income of a region, the higher the population emigration rate will be^58^. Therefore, we take the expected income ratio (EIP) between the average wage of employees in Xi’an and the average wage of employees in China as the wage income impact factor (IF) and take it as one of the indicators affecting the mechanical emigration rate of population (Eq. 8). In addition, residential environment factors (LF) characterized by population density (PD) are also considered as one of the indicators affecting the mechanical emigration rate of population (Eq. 9).
3. Population transformation. Population transformation refers to the transformation process from a floating population to a permanent population within a city, which is described by Eqs. (10) and (11). In this model, the process is affected by the population transformation velocity (SF). There are many factors that affect the speed of demographic transition, such as housing prices, employment rates, etc. To keep the model concise and efficient, only the representative employment rate (JER) is selected as the index of population transformation speed (Eq. 10). The employment rate is affected by various factors, so we set the employment coefficient (EC) to correct the calculation result of the employment rate (Eq. 11).
  1 $$NI = x_{1} \cdot GDPF + x_{2} \cdot MF + x_{3} \cdot ERF$$
  2 $$GDPP = x_{4} \cdot FR + x_{5} \cdot RSR + x_{6} \cdot SLR$$
  3 $$SLR = x_{7} \cdot RDP + x_{8} \cdot STR$$
  4 $$MF = x_{9} \cdot ADA + x_{10} \cdot ABA$$
  5 $$ERF = x_{11} \cdot ER + x_{12} \cdot ENP + x_{13} \cdot SRP + x_{14} \cdot TRP$$
  6 $$NO = x_{15}$$
  7 $$MO = x_{16} \cdot LF + x_{17} \cdot IF$$
  8 $$IF = x_{18} \cdot EIP$$
  9 $$LF = x_{19} \cdot PD$$
  10 $$SF = x_{20} \cdot JER$$
  11 $$EC = x_{21}$$

**Table 1:** Meaning of specific abbreviations in the formulas.

Variables	Details
NI	The number of people moving in
GDPF	Economic impact factors affecting population migration
MF	Health infrastructure influencing factors of population migration
ERF	Educational infrastructure influencing factors of population migration
GDPP	Economic growth rate
FR	Growth rate of fixed assets
RSR	Real wage growth rate
SLR	Growth rate of science and technology level
RDP	R&D capital growth rate
STR	Growth rate of the proportion of scientific expenditure
ADA	Growth rate of doctors per capita
ABA	Growth rate of beds per capita
ER	Growth rate of proportion of education expenditure
ENP	Growth rate of teaching facilities
SRP	Growth rate of the number of students in school per capita
TRP	Growth rate in the number of teachers per capita
NO	Natural emigration rate
MO	Mechanical emigration rate of population
LF	Influencing factors of living environment on mechanical emigration rate of population
IF	Influencing factors of wage income on mechanical emigration rate of population
EIP	Expected income ratio
PD	The population density
SF	Factors affecting the speed of population transformation
JER	The employment rate
EC	The coefficient of employment

#### Parameter optimization by MOLsOA

The lioness optimization algorithm (LsOA) is an optimization algorithm inspired by lioness team hunting and elite hunting. In group hunting mode, the lioness population gradually fanned out towards the prey, and eventually, the target was captured by the lionesses located near the “central” circle. Another hunting mode of the lioness population is elite hunting, meaning that the top lioness of the population occasionally hunt alone, a mode of survival under the theory of survival of the fittest. A detailed implementation of LsOA can be found in the research of Li et al.^8^. Because LsOA is a single objective optimization algorithm, it can’t optimize multiple objectives simultaneously, so we improved it and proposed a multi-objective lioness optimization algorithm (MOLsOA). The specific improvement strategies are as follows.

1. Strategy for updating the position of the lioness.

In contrast to LsOA, MOLsOA does not adopt the position update strategy of a single-objective optimization algorithm when updating the lioness’s position. Instead, MOLSOA sets a fresh position update strategy in the context of increasing population diversity:

12 $$\vec{X} = \left\{ {\begin{array}{*{20}l} {\vec{X}_{new} {,}\;{\text{ if }}\;f(\vec{X}_{new} ) \prec \, f(\vec{X}_{old} ) \, } \\ {\vec{X}_{new} {,}\;{\text{ if}}\; \, f(\vec{X}_{old} ){\text{ and }}f(\vec{X}_{new} )\;{\text{ do}}\;{\text{ not }}\;{\text{dominate}}\;{\text{ each}}\;{\text{ other}}\; \, \cap \; \, rand < P_{threshold} } \\ {\vec{X}_{old} {, }\;{\text{if }}\;f(\vec{X}_{old} ) \prec \, f(\vec{X}_{new} ) \, } \\ \end{array} } \right.$$

where, $\vec{X}$ represents the position of an individual after updating, $\vec{X}_{new}$ represents the new position after performing a search, $\vec{X}_{old}$ represents the position before performing the optimization operation, $rand$ represents a random number between 0 and 1, and $P_{threshold}$ represents the threshold. The larger the value of $P_{threshold}$, the more likely the possibility of population renewal and the greater the population diversity.

2. Create an external elite repository (REP)

Unlike single-objective optimization algorithms, in multi-objective optimization there is normally no situation where one individual is superior to all others. Moreover, it is not possible to rank all the individuals in the population according to the appropriate value, which fails to select the superior individuals from the population, the elite lionesses of MOLsOA. The creation of REP solved this problem. After the population initialization is completed, the multi-objective function values of each individual in the population are calculated and non-inferior individuals are selected to join the REP. The elite lionesses will be randomly selected from the REP. ​The significance of REP is to preserve high-quality solutions in the population, to address the difficulty of updating individual locations and the slow convergence rate of the population, and to continuously provide adequate exploration directions for subsequent evolution, enabling efficient search in the decision space.

At each iteration, the REP is updated. When a new individual appears in the REP that can dominate an individual, the original individual is stored in the REP and the dominated individual is removed. The adaptive grid crowding strategy is used to deal with the case when the number of individuals in REP exceeds the maximum storage capacity^49^.

The pseudo-code of MOLsOA is given in Algorithm 1, and the steps of how to use MOLsOA to optimize the critical parameters of the urban population dynamics model are detailed below.

Step 1: Initializing the population, relevant parameters, and $REP$.

First, the urban population prediction model involves a myriad of variables. Population and GDP are chosen as the optimization objectives of MOLsOA based on the availability of data and the representability of variables. Population and GDP data from 2000 to 2018 are used to participate in the optimization. Second, the decision variables of MOLsOA are the 21 parameters to be optimized introduced in Sect. 3.2.1, namely $[x_{1} ,x_{2} ,x_{3} , \ldots ,x_{21} ]$, $x_{i} \in [0.01,1]$.

Step 2: Non-inferior solutions in the population are added to $REP$.

In this step, the multi-objective function value of each individual in the population is calculated, that is, the fitness value of each individual. To accurately model future trends in urban populations, predictive models must achieve high fitting accuracy when simulating historical data. For this purpose, the absolute percentage error (APE) between simulated data and actual data is taken as the optimization objective of MOLsOA. By looking at historical data, it can be seen that the population growth rate in various regions has increased significantly in recent years, and the population development has presented a new situation unprecedented in history. Only by absorbing and reflecting on the new situation can the prediction model accurately predict the future trends of the urban population. Therefore, when designing optimization objectives, the APE of population and GDP are designed with different weights each year, and the mean absolute percentage error (MAPE) with influence weights is obtained, which is taken as the final optimization objective, as shown in Eqs. (13) and (14).

13 $$fitness\_PT = \frac{1}{n}\sum\limits_{i = 1}^{n} {Q_{i} \left| {\frac{{FPT_{i} - RPT_{i} }}{{RPT_{i} }}} \right|}$$

14 $$fitness\_GDP = \frac{1}{n}\sum\limits_{i = 1}^{n} {P_{i} \left| {\frac{{FGDP_{i} - RGDP_{i} }}{{RGDP_{i} }}} \right|}$$

where, $i = 1,\;2,\;3,\ldots,n$ and $n = 19$ represents the period from 2000 to 2018, $RPT_{i}$, $RGDP_{i}$ represents the actual annual data of the population from 2000 to 2018, $FPT_{i}$, $FGDP_{i}$ represents the annual data of the population from 2000 to 2018 obtained by SD model simulation, $Q_{i}$, $P_{i}$ represents the influence weight corresponding to APE of population and GDP from 2000 to 2018.

Step 3: In team hunting mode, the center circle is constructed to capture prey.

According to the lioness's hunting pattern, prey should be located in the heart of the pride. As a result, the central circle can help the lionesses locate their prey more accurately. The center circle consists of $t$ lionesses randomly selected from $REP$ and their average values (Eq. 15). Since the location of the prey in the search space is unknown, it is assumed that the prey can be located at any of the locations that make up the center circle, and each location is assumed to be chosen with probability $\frac{1}{t}$.

15 $$E\_Team = \left[ {\vec{X}_{a} ;\vec{X}_{b} ;\vec{X}_{...} ;\vec{X}_{t} ;\vec{X}_{ave} } \right]$$

where, $\vec{X}_{j}$ and $j = a,b,\ldots,t$ represents the position of $t$ lionesses, $\vec{X}_{ave}$ represents the average of all lionesses.

Then, based on the central circle, the individual positions of the population are updated according to the synchronized walking strategy in LsOA to capture prey.

Step 4: In elite hunting mode, an elite matrix is constructed to capture prey.

Elite lionesses are used as group agents in the capture of prey. At this time, the lionesses switched from team hunting to elite hunting, relying on elite lionesses to capture prey. The top $r$ lionesses ranked by fitness value in the last iteration are selected, and their average value is called the top lioness (Eq. 16), which can avoid the problem of falling into local optimum only by relying on a single elite position. The position of the top lioness is replicated into $n$ rows to form the elite matrix (Eq. 17).

16 $$Top\_lioness\_pos = \frac{{\vec{X}_{a} + \vec{X}_{b} + \vec{X}_{...} + \vec{X}_{r} }}{r}$$

17 $$Elite\_lioness = \left[ {\begin{array}{*{20}c} {X_{1,1}^{I} } & {X_{1,2}^{I} } & \ldots & {X_{1,d}^{I} } \\ {X_{2,1}^{I} } & {X_{2,2}^{I} } & \cdots & {X_{2,d}^{I} } \\ \vdots & \vdots & \vdots & \vdots \\ \vdots & \vdots & \vdots & \vdots \\ \vdots & \vdots & \vdots & \vdots \\ {X_{n,1}^{I} } & {X_{n,2}^{I} } & \ldots & {X_{n,d}^{I} } \\ \end{array} } \right]_{n \times d}$$

where, $\vec{X}_{m}$ and $m = a,b,\ldots,r$ in Eq. (16) represents the position vectors of the $r$ lionesses with the highest fitness value in the last iteration, $\vec{X}^{I}$ in Eq. (17) represents the position vector of the top lioness, $d$ represents the number of parameters to be optimized, and its value is 21, $n$ is the size of the population.

Then, based on $Elite\_lioness$, the individual positions of the population are updated according to the phase focusing mechanism in LsOA to capture prey.

Step 5: The population position was updated according to our proposed lioness individual position updating strategy (Eq. 12) and $REP$ was corrected by comparison.
Step 6: Drifting fish aggregating devices (FADs) of the LsOA were used to update the current population position.
Step 7: Determine whether the algorithm is complete.

In this step, it is necessary to determine whether the outcome of this round satisfies the end condition of the iteration. If yes, $REP$ will be output and the algorithm will end. If not, return to Step 2 to continue updating and optimizing.
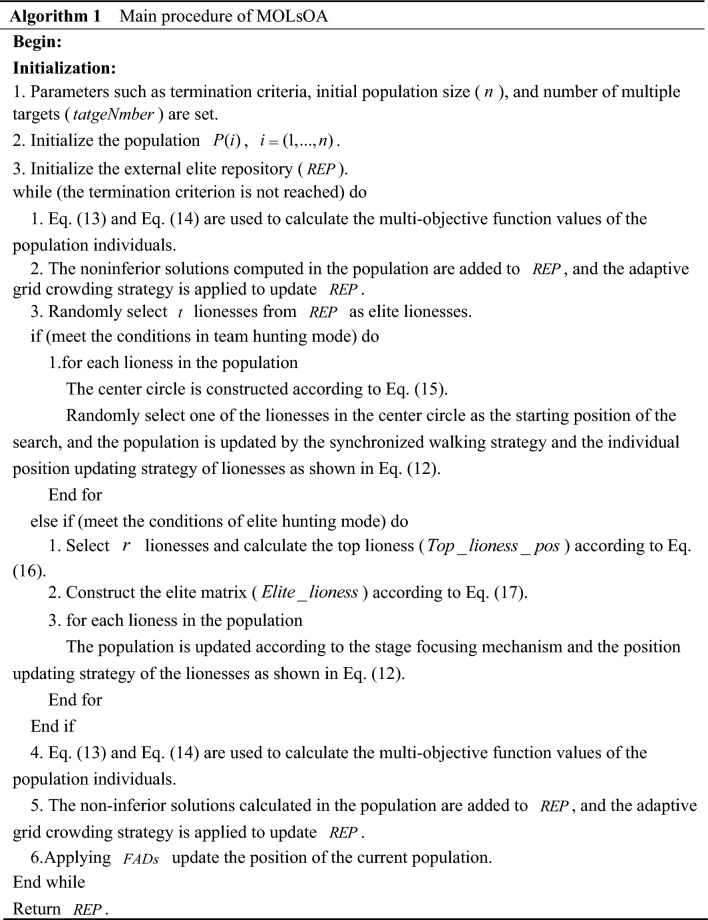


#### Performance analysis of MOLsOA

1. Test functions, comparison algorithms, and evaluation indicators

To investigate the performance of MOLsOA, we chose to use the eight multimodal multi-objective test functions (MMFs) as the test function for the experiment. MMFs are one of the most representative test problems. They are designed by mirroring the original PS to form multiple equivalent subsets. Additional information regarding MMFs can be found at^59^.

In addition, we also selected eight multi-objective optimization algorithms for comparison in this experiment, namely ANSGAIII^60^, BiGE^61^, MOEA/D^62^, MOHCO^63^, MOPSO^64^, NSGAII^65^, SPEA2^66^ and WOF^67^. The ANSGAIII and MOPSO are Pareto dominance-based representative multi-objective evolutionary algorithms (MMEAs). MOEA/D and NSGAII are classic MOEAs. WOF and BiGE are two state-of-the-art MMEAs. For the purpose of unbiased comparison, the population size is set to 100, the maximal number of evaluations is set to 80,000 for all the algorithms. All the experiments are carried out 30 times.

Finally, we evaluated the running results through three evaluation indicators, namely Cover Rate (CR)^59^, Inverted Generational Distance in the decision space (IGDX)^68^, and Pareto Sets Proximity (PSP)^59^ indicators. The specific descriptions of these three indicators are as follows:

① The CR indicator is perhaps one of the most popular indicators, it can show overlap ratio between true Pareto-optimal set (PS) and obtained PS. Larger CR values are desirable. The CR defined as:
  18 $$CR = \left( {\prod\limits_{l = 1}^{n} {\delta_{l} } } \right)^{1/2n}$$
  19 $$\delta_{l} = \left\{ {\begin{array}{*{20}c} 1 & {V_{l}^{\max } = V_{l}^{\min } } \\ 0 & {v_{l}^{\min } \ge \left. {V_{l}^{\max } } \right\|v_{l}^{\max } \le V_{l}^{\min } } \\ {\left( {\frac{{\min \left( {v_{l}^{\max } ,V_{l}^{\max } } \right) - \max \left( {v_{l}^{\min } ,V_{l}^{\min } } \right)}}{{V_{l}^{\max } - V_{l}^{\min } }}} \right)^{2} } & { \, otherwise \, } \\ \end{array} } \right.$$
  where $n$ is the dimensionality of decision space; $v_{l}^{\max }$ and $v_{l}^{\min }$ are respectively the maximum and minimum of obtained PS for the $l{\text{th}}$ variable; $V_{l}^{\max }$ and $V_{l}^{\min }$ are the maximum and minimum of the true PS for the $l{\text{th}}$ variable.
② However, CR cannot show the diversity and convergence of the obtained solutions. So, the IGDX indicator is proposed, it can evaluate how well the obtained solution set approximates the true solution set in the decision space. The IGDX defined as:
  20 $$IGDX\left( {O,P^{*} } \right) = \frac{{\sum\limits_{{v \in P^{*} }} d (v,O)}}{{\left| {P^{*} } \right|}}$$
  where $P^{*}$ is a set of uniformly distributed points along the true PS. $O$ is a set of obtained solutions. The IGDX can be calculated as the average distance from $P^{*}$ to $O$.
③ The PSP indicator is combined from CR and IGDX. PSP can not only reflect the convergence of obtained PS, but also represent overlap ratio between true PS and obtained PS. Larger PSP values are desirable. The PSP defined as:
  21 $$PSP = \frac{CR}{{IGDX}}$$
  where $CR$ is the cover rate, which has been defined in Eq. (18). $IGDX$ is the inverted generational distance in the decision space, which has been defined Eq. (20). Larger $PSP$ values are desirable.

2. Results of MOLsOA and discussion.

**Figure 3:**
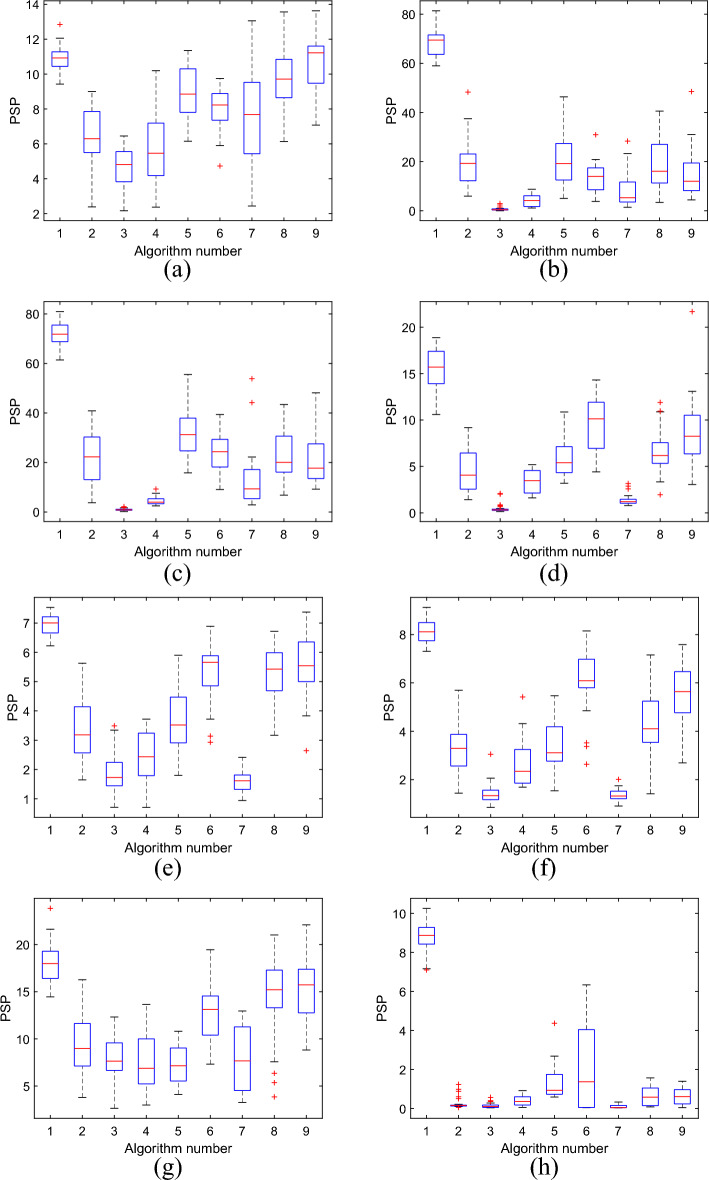
The box-plots of PSP values of different algorithms on eight test functions. (**a**) MMF1; (**b**) MMF2; (**c**) MMF3; (**d**) MMF4; (**e**) MMF5; (**f**) MMF6; (**g**) MMF7; (**h**) MMF8.

The statistical results of PSP values are shown in Fig. 3 by box-plots. The numerals on the horizontal axis of each plot indicate the following algorithms: 1 = MOLsOA, 2 = ANSGAIII, 3 = BiGE, 4 = MOEAD, 5 = MOHCO, 6 = MOPSO, 7 = NSGAII, 8 = SPEA2, and 9 = WOF.

In the boxplot, each box has five lines, representing five statistics from top to bottom: maximum, upper quartile, mean, lower quartile, and minimum. Since PSP index requires the larger the better, and the algorithm has the characteristics of robustness, the ideal experimental results should require a relatively concentrated data distribution based on the pursuit of the maximum value, that is, the box in the figure should be high and as flat as possible. As can be seen from the Fig. 3, the mean PSP values for MOLsOA are highest on all test functions, and the distribution of PSP values is relatively concentrated on test functions except MMF4 and MMF8, indicating that MOLsOA performs better and is relatively stable compared with other algorithms. In addition, WOF ranked second on MMF1, MMF5 and MMF7. MOHCO ranked second on MMF2 and MMF3. MOPSO ranked second on MMF4, MMF6 and MMF8. These three algorithms are a somewhat better than ANSGAIII, BiGE, MOEAD, NSGAII and SPEA2. The reason why ANSGAIII and MOEAD perform poorly on all test functions is that these methods consider only the crowding distance in the objective space. The PSP values of WOF, MOHCO and MOPSO vary greatly, resulting in a large variance of the overall data, and the experimental results are not stable.

Tables 2 and 3 are the CR and IGDX values of the different algorithms on the eight benchmark functions, respectively. It can be seen from Tables 2 and 3 that compared with other algorithms, MOLsOA has achieved the optimal results on the indicator values of the test functions. For the CR indicator, according to the Friedman mean rank, MOLsOA performed the best and ranked first overall, outperforming MOPSO, WOF, MOHCO, SPEA2, ANSGAIII, MOEAD, NSGAII and BiGE by 18%, 25%, 32%, 38%, 58%, 69%, 70% and 82%. For the IGDX indicator, according to the Friedman mean rank, MOLsOA also performed the best, ranking first overall, which was in turn superior to SPEA2, WOF, MOPSO, MOHCO, ANSGAIII, MOEAD, NSGAII and BiGE by 250%, 250%, 275%, 300%, 450%, 638%, 700% and 738%. The above results once again prove the superior performance of MOLsOA.

**Table 2:** IGDX values of different algorithms.

Function		MOLsOA	ANSGAIII	BiGE	MOEAD	MOHCO	MOPSO	NSGAII	SPEA2	WOF
MMF1	Ave	**0.0923**	0.1512	0.2006	0.1742	0.1145	0.1266	0.1511	0.1028	0.0954
	Std	0.0064	0.0435	0.0434	0.0588	0.0179	0.0221	0.0672	0.0173	0.0149
MMF2	Ave	**0.0147**	0.0616	0.5832	0.2349	0.0601	0.0972	0.1638	0.0734	0.0975
	Std	0.0012	0.0325	0.1845	0.1151	0.0363	0.0563	0.1112	0.0536	0.0547
MMF3	Ave	**0.0140**	0.0521	0.3997	0.1678	0.0329	0.0478	0.0976	0.0473	0.0578
	Std	0.0010	0.0321	0.1044	0.0346	0.0103	0.0192	0.0550	0.0212	0.0238
MMF4	Ave	**0.0666**	0.2284	0.9306	0.2861	0.1885	0.1169	0.4663	0.1686	0.1308
	Std	0.0109	0.0895	0.2457	0.0820	0.0561	0.0459	0.1110	0.0653	0.0512
MMF5	Ave	**0.1440**	0.2884	0.4367	0.3834	0.2765	0.1948	0.4579	0.1890	0.1819
	Std	0.0075	0.0833	0.0971	0.1440	0.0665	0.0465	0.0683	0.0353	0.0411
MMF6	Ave	**0.1230**	0.2653	0.4142	0.3103	0.2696	0.1681	0.4167	0.2179	0.1818
	Std	0.0065	0.0583	0.0532	0.0614	0.0501	0.0422	0.0350	0.0538	0.0413
MMF7	Ave	**0.0538**	0.0962	0.1173	0.1311	0.1016	0.0706	0.1353	0.0741	0.0664
	Std	0.0046	0.0330	0.0458	0.0497	0.0177	0.0137	0.0523	0.0337	0.0165
MMF8	Ave	**0.1130**	2.0652	2.5827	1.7683	0.8381	1.5254	2.8367	1.4554	1.4800
	Std	0.0107	0.5798	0.5495	0.5969	0.3123	1.3587	0.5251	0.7426	0.7569
Friedman mean rank		1.00	5.50	8.38	7.38	4.00	3.75	8.00	3.50	3.50
Rank		1	5	8	6	4	3	7	2	2

The Ave index value of each test problem is in bold.

**Table 3:** CR values of different algorithms.

Function		MOLsOA	ANSGAIII	BiGE	MOEAD	MOHCO	MOPSO	NSGAII	SPEA2	WOF
MMF1	Ave	**0.9986**	0.9062	0.8881	0.8694	0.9931	0.9893	0.9390	0.9714	0.9812
	Std	0.0012	0.0588	0.0759	0.0762	0.0083	0.0126	0.0820	0.0235	0.0180
MMF2	Ave	**1.0000**	0.9588	0.2938	0.7670	1.0000	**1.0000**	0.7932	0.9508	1.0000
	Std	0.0000	0.0440	0.1268	0.1464	0.0000	0.0000	0.1272	0.0556	0.0000
MMF3	Ave	**1.0000**	0.8831	0.3370	0.7148	1.0000	**1.0000**	0.8054	0.9116	0.9985
	Std	0.0000	0.0920	0.0975	0.1182	0.0000	0.0000	0.1080	0.0739	0.0084
MMF4	Ave	**0.9994**	0.8582	0.3410	0.8501	0.9860	0.9893	0.5724	0.9469	0.9712
	Std	0.0009	0.1274	0.1095	0.1079	0.0243	0.0152	0.0563	0.0628	0.0418
MMF5	Ave	**0.9988**	0.9139	0.7489	0.7940	0.9375	0.9923	0.7133	0.9678	0.9683
	Std	0.0010	0.0710	0.1307	0.1199	0.0577	0.0089	0.0870	0.0322	0.0324
MMF6	Ave	**0.9985**	0.8115	0.5725	0.7667	0.8827	0.9756	0.5538	0.8920	0.9519
	Std	0.0013	0.1059	0.0831	0.1121	0.0847	0.0438	0.0607	0.1015	0.0552
MMF7	Ave	**0.9603**	0.8119	0.8010	0.8571	0.7246	0.8619	0.8825	0.9520	0.9550
	Std	0.0448	0.0813	0.0914	0.0673	0.1100	0.0917	0.0918	0.0567	0.0278
MMF8	Ave	**0.9855**	0.4011	0.3357	0.5749	0.8604	0.5918	0.2479	0.5963	0.6578
	Std	0.0135	0.1810	0.1820	0.1933	0.0967	0.3734	0.1535	0.2419	0.2287
Friedman mean rank		8.88	3.75	1.63	2.75	6.00	7.25	2.63	5.50	6.63
Rank		1	6	9	7	4	2	8	5	3

The Ave index value of each test problem is in bold.

In conclusion, MOLsOA obtains the best distribution in decision space and in objective space on all the eight test problems. It shows that it is competitive with other algorithms in solving multi-objective optimization problems, and all of the above verify the effectiveness of MOLsOA.

## Empirical test

Xi’an, an important central city in western China approved by the State Council, is one of China's megacities and a representative city of the country’s rapid economic development. We empirically test our proposed urban population prediction system model using relevant data from Xi'an, which has some reference value to demonstrate the practicability of the model.

The space boundary of the system is limited to Xi'an city, the time boundary is limited to 2000–2050 (based on the year 2000), and the simulation time step is 1 year.

### The current population situation in Xi’an

**Figure 4:**
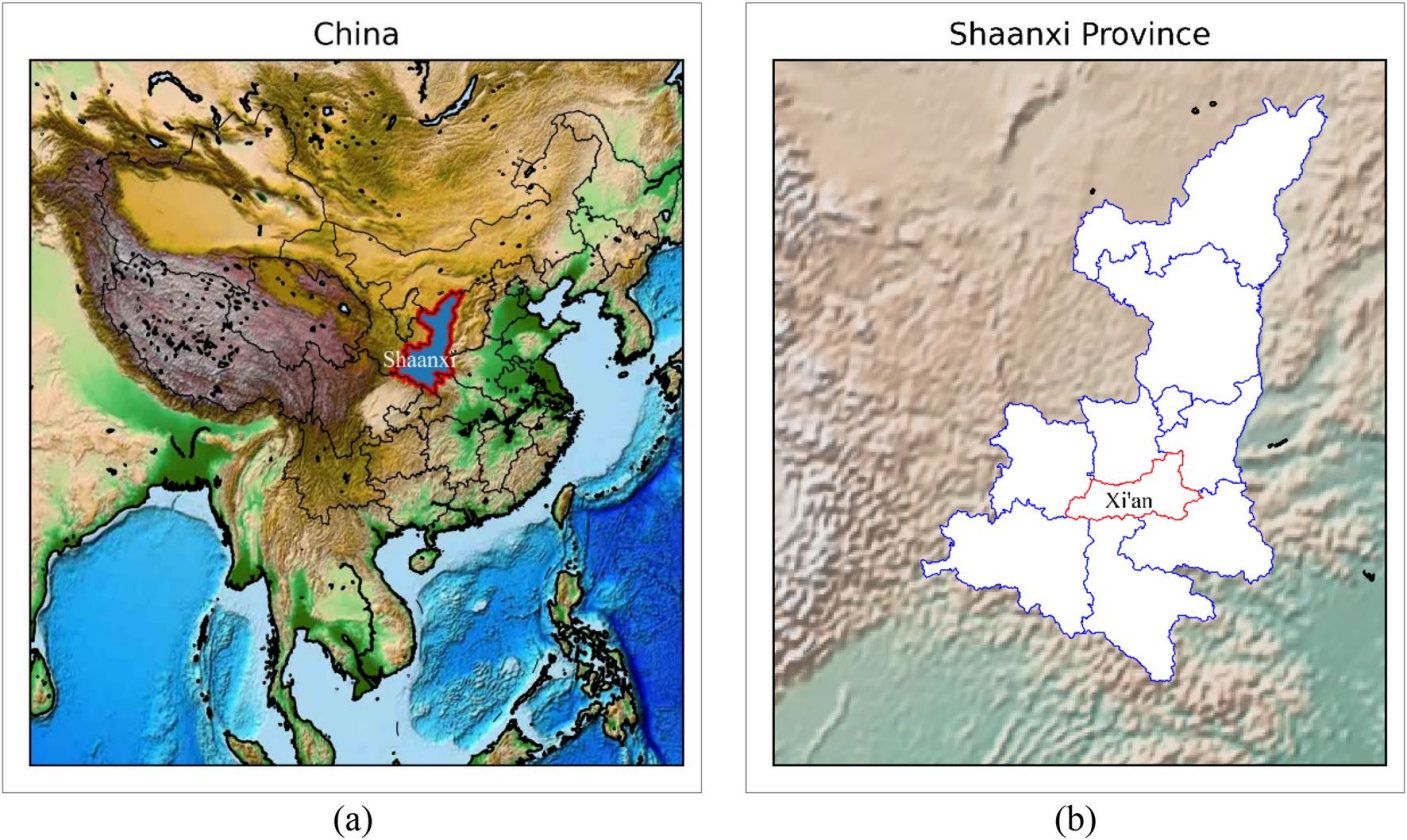
Regional map of Xi’an. (**a**) Regional map of Shaanxi Province, China; (**b**) regional Map of Xi’an, Shaanxi Province.

**Figure 5:**
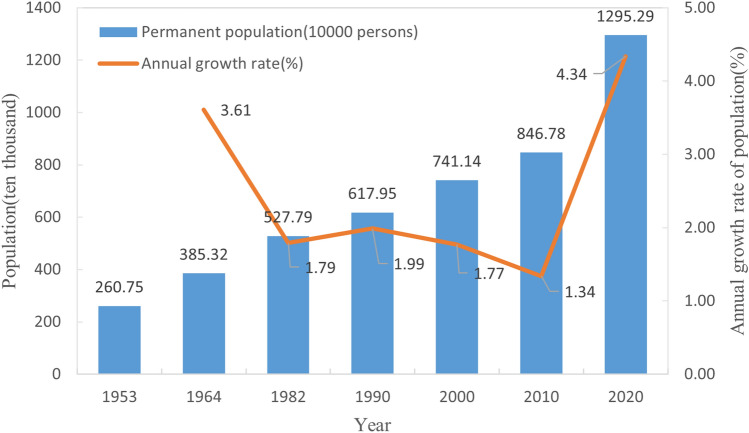
The permanent population and average annual growth rate of Xi’an in previous population censuses.

Xi’an is an essential central city in western China approved by the State Council and one of China’s megacities. The regional map of Xi’an is shown in Fig. 4. By the end of 2020, the total area of Xi’an (including Xixian New Area) is 10,752 square kilometers, the permanent population is 12952900, and the GDP is 1002.039 billion yuan. Figure 5 shows the population trend of Xi’an in all previous censuses (1953–2020).

According to Fig. 5 the post-liberation population growth in Xi’an experienced three main stages: The first stage was from 1953 to 1982. The total population grew rapidly, with an average annual growth rate of 3.61 percent. The second stage ran from 1982 to 2010. The average annual growth rate of Xi’an’s population is stable at [1.34%, 1.99%]. The third stage is from 2010 to the present. The total population grew rapidly, with an average annual growth rate of 4.34 percent.

### Data sources

The population of Xi’an is simulated based on the relationship between the variables in the aforementioned stock flow graph. In this paper, the historical data for each parameter is mainly obtained from the Xi’an Statistical Yearbook, Shaanxi Statistical Yearbook, and China Urban Statistical Yearbook. Parameters such as natural population growth rate, registration of the enterprise, national average employment rate, fixed asset investment growth rate, average wage growth rate, and growth rate of the proportion of scientific expenditure are expressed by using the With Lookup function. In this paper, the data for the With Lookup function from 2000 to 2019 are taken from the aforementioned almanacs. Data from 2020 to 2050 are predicted based on the overall domestic or current development of Xi’an.

### MOLsOA optimization results

The population size is set to 100 and the maximum number of iterations is 8000. After many tests, the influence weight $Q_{i}$ and $P_{i}$($i = 1,2,3,\ldots,19$) are set as follows: except for $Q_{18} = Q_{19} = 1.5$, all the others are 1, that is, the influence weight of the population in 2017 and 2018 is 1.5, and the influence weight of the population and GDP in other years is 1. In addition, the number of elite lionesses forming the center circle in the team hunting mode ($t$) is set as: $t = 4$, and the number of lionesses used to calculate the position of the top lioness in the elite hunting mode ($r$) is set as: $r = 3$.

**Figure 6:**
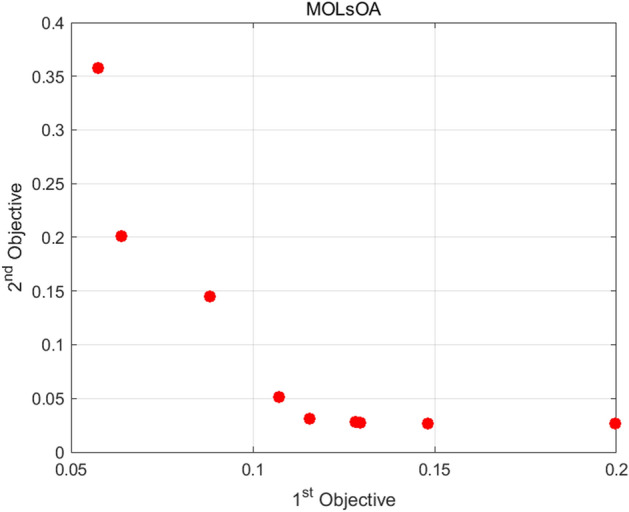
Evolution result of MOLsOA.

When the training reaches the maximum number of iterations, MOLsOA find 9 sets of solutions. The target values for the total population and GDP corresponding to each group of solutions are [0.107123; 0.051149], [0.129460; 0.027650], [0.115561; 0.030845], [0.063735; 0.201496], [0.148058; 0.026829], [0.128285; 0.028239], [0.088260; 0.145193], [0.057350; 0.357864], [0.199767; 0.026357]. After conversion, the MAPE with impact weights for the simulated values of population and GDP and the actual values in the 9 sets of solutions are: [10.7123%; 5.1149%], [12.9460%; 2.7650%], [11.5561%; 3.0845%], [6.3735%; 20.1496%], [14.8058%; 2.6829%], [12.8285%; 2.8239%], [8.8260%; 14.5193%], [5.7350%; 35.7864%], [19.9767%; 2.6357%]. Figure 6 shows the specific cases of the nine sets of solutions. The horizontal axis $1{\text{st}}\;Objective$ represents the target value of the population, and the vertical axis $2{\text{st}}\;Objective$ represents the target value of GDP.

To select the optimal solution from multiple groups of solutions, the following principles are designed in this paper.

Principle 1: The smaller the value of both objectives, the better.

Principle 2: The APE of each year's simulated data for both targets is as stable as possible.

Based on the above principles, [0.115561; 0.030845] is selected as the optimal solution from the 9 groups of solutions obtained through optimization, and the optimal individual values from $x_{1}$ to $x_{21}$ corresponding to this solution are shown in Table 4. The simulation results are described in detail in the history check section.

**Table 4:** The results of parameter optimization.

Parameter	Value	Parameter	Value	Parameter	Value
$x_{1}$	0.230567	$x_{8}$	0.291284	$x_{15}$	0.01
$x_{2}$	0.010593	$x_{9}$	0.014384	$x_{16}$	0.01
$x_{3}$	0.01	$x_{10}$	0.02722	$x_{17}$	0.01
$x_{4}$	0.01	$x_{11}$	0.01	$x_{18}$	0.089249
$x_{5}$	1	$x_{12}$	0.01	$x_{19}$	0.01
$x_{6}$	0.012305	$x_{13}$	0.188478	$x_{20}$	0.498094
$x_{7}$	0.385782	$x_{14}$	0.122732	$x_{21}$	0.01

### Model simulation

Substituting the optimization results obtained by MOLsOA in the above section into the variable equations (Eqs. 1–11), a complete urban population prediction system dynamics model with Xi’an population as the research object can be obtained, and the system dynamics software Vensim is used for simulation. To verify the effectiveness of the model, we performed visual inspection, historical inspection, and stability test.

1. Visual inspection: Visual inspection includes checking the correctness of variable settings, causality, and model structure of the system model. We comprehensively considered economic, educational, medical, and other factors to analyze the urban population prediction system, and used Vensim for model testing and unit testing of the prediction model. The results show that the causal relationship of the model is reasonable, there are no abnormal results, and the operation process of the actual system can be better reproduced.
2. Historical inspection: Historical inspection after the model design is completed, the simulation value of the model is compared with the actual deal to verify the validity of the model. We choose population and GDP as representative variables for the history test. The historical test interval is 2000–2018 years. The results are shown in Table 5. The historical test results show that the error percentage between the simulated value and the actual value is between − 10% and 10%. According to the study of Lewis^69^, the test results are acceptable, which also indicates that the sensitivity and robustness of our model are low, and the development and change of the system can be studied by adjusting different variables to set various scenarios.

**Table 5:** Comparison of simulated and historical values of population and GDP.

Year	Population/(10^2^)			GDP/(10^6^ Yuan)		
	True value	Simulation	Error	True value	Simulation	Error
2005	80,681	80,845	− 0.00203	127,014	130,942.378	− 0.03093
2006	82,252	83,140	− 0.01079	147,368	149,878.538	− 0.01704
2007	83,054	84,575	− 0.01831	176,373	184,937.284	− 0.04856
2008	83,752	86,832	− 0.03678	219,004	230,689.964	− 0.05336
2009	84,346	89,309	− 0.05884	272,408	282,913.833	− 0.03857
2010	84,741	91,623	− 0.08122	324,149	333,003.751	− 0.02732
2011	85,134	92,671	− 0.08853	386,421	398,488.830	− 0.03123
2012	85,529	92,332	− 0.07954	436,610	442,460.209	− 0.01340
2013	85,881	91,952	− 0.07070	488,413	492,327.961	− 0.00802
2014	86,275	90,677	− 0.05102	549,264	571,557.544	− 0.04059
2015	87,056	92,332	− 0.06061	580,120	559,237.366	0.03600
2016	88,321	92,123	− 0.04305	625,718	662,227.714	− 0.05835
2017	96,167	94,238	0.02005	719,210	724,678.345	− 0.00760
2018	100,037	93,261	0.06774	834,986	817,378.558	0.02109
3. Stability test: It refers to the selection of different simulation time steps for testing to verify the overall stability of the model. Three simulation time steps of one year, half year, and quarter year are selected for testing, and the simulation results are shown in Fig. 7. The results show that when the simulation time step is varied, the overall trend of the model is the same, although the simulation results for individual years are different. The results show that the model has passed the stability test, that is, the model is stable and feasible.

**Figure 7:**
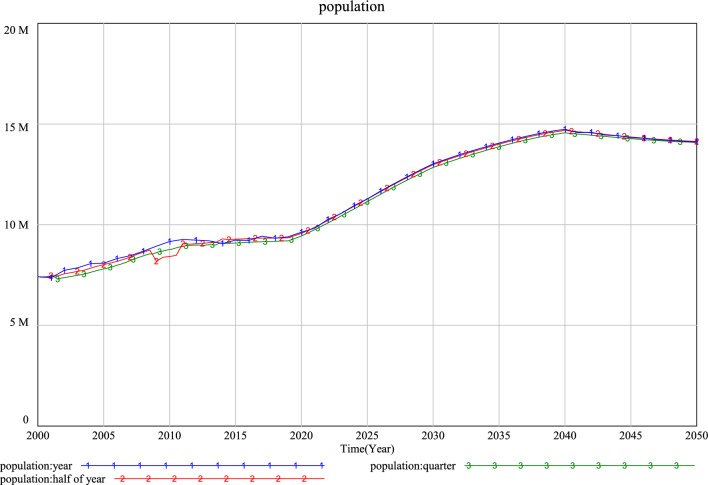
Asynchronous simulation value test results.

From the above tests, it can be seen that the system dynamics model we have developed has excellent simulation capabilities and can better reflect the actual development trends of the Xi’an population. It is suitable for subsequent research using scenario analysis methods.

## Policy scenario analysis

### Scenario analysis and dynamic simulation

We will use scenario analysis to observe population changes under different policies. Since the population prediction system is a vast system affected by myriad factors, we only select a few policy factors for scenario analysis.

The growth of a population comprises two distinct components: natural and mechanical growth. Natural growth denotes the increase resulting from birth rates minus death rates, while mechanical growth refers to the elevation from in-migration over out-migration^70^. In light of these two types of growth, we propose five policies, namely, fertility, employment, science and technology, healthcare and education policies. The reasons for choosing these policies are as follows.

1. Fertility policy plays a critical role among numerous policies impacting natural population growth. Given China's aged population and declining birth rate, this policy constitutes a vital aspect of population policy. Consequently, investigating the influence of this policy on population growth carries significant research merit.
2. Among the multiple policies that have an impact on the mechanical growth of the population, we have chosen to study employment, science and technology, healthcare and education policies. Firstly, China-a developing country with high population density-has long been under extensive employment pressure, and the gloomy employment prospects have made job availability and wage income crucial considerations influencing population mobility^71^. To explore population movements under different wage incomes and employment opportunities, we set up and study an employment policy that reflects both. Secondly, scientific and technological innovation constitutes an integral aspect of China’s overall national development, reflecting the urban advancement standard. Enhancing such policies would enable local authorities to lure innovative talent and induce population movements. Therefore, it is of some significance to explore the impact of science and technology policies on the population. Finally, healthcare and education are crucial components of public services that are of vital interest to all residents. Promoting the development of healthcare and education policies is critical in improving the livability of cities, attracting a mobile population, and ultimately mitigating the aging population and fostering population growth. Quantitatively exploring the impact of health and education policies on the population holds significant research value.

Based on the above analysis, we selected the scenario analysis variables include: natural population growth rate, average wage growth rate, coefficient of employment, the growth rate of R&D investment, rate of increase in the number of beds, rate of increase in the number of doctors, the growth rate of the number of schools, the growth rate of teachers, the growth rate of the number of students in school. The control parameters of each policy design are shown in Table 6. The benchmark scenario is based on the current development and future development plans of Xi’an, mainly based on the Outline of the 14th Five-Year Plan and 2035 Long-term Goals for the National Economic and Social Development of Xi’an^72^, the 14th Five-Year Plan of Xi’an Education Development^73^, and the 14th Five-Year of Xi’an Health Development Plan^74^, etc. In addition to designing the baseline scenario, we combine the nine moderating variables to build five single policy scenarios and two combined policy scenarios. Among them, five single policy scenarios include low speed mode and high speed mode, respectively. Specifically, the single policy scenarios are birth policy scenario (scenarios A1 and B1), employment policy scenario (scenarios A2 and B2), science and technology policy scenario (scenarios A3 and B3), healthcare policy scenario (scenarios A4 and B4), and education policy scenario (scenarios A5 and B5). Scenarios A1, A2, A3, A4 and A5 are the low speed modes of each single policy scenario, while scenarios B1, B2, B3, B4 and B5 are the highspeed modes of each single policy scenario. Policy mix scenarios include low-speed development scenario (scenario C) and high-speed development scenario (scenario D). Low-speed development scenario (scenario C) uses the parameter settings of five single policy scenarios in low speed mode (scenario A1, A2, A3, A4 and A5), and high-speed development scenario (scenario D) uses the parameter settings of five single policy scenarios in highspeed mode (scenario B1, B2, B3, B4 and B5). The specific description of the scenarios is given below.

1. The setting of the single policy scenario is to analyze the influence of various policy factors on regional population development in low speed mode and high speed mode, and to study which policy will play a key or essential role in the future population development.
2. When formulating the population development plan, the government is more inclined to use “combination boxing” and through the coordination of various policy measures and regulatory means, amplify the policy implementation and give better play to the combined effect of “1 + 1 > 2”. Our policy mix scenario setting is based on this idea.

**Table 6:** Parameters settings for different scenarios.

Scenario name	Scenario code and description		The model parameters controlled in the scenario
Benchmark scenario	O		–
Single policy scenarios	A1	Birth Policy Scenario (Low-speed)	Natural population growth rate
	B1	Birth Policy Scenario (High-speed)	
	A2	Employment Policy Scenario (Low-speed)	Average wage growth rate and coefficient of employment
	B2	Employment Policy Scenario (High-speed)	
	A3	Science and Technology Policy Scenario (Low-speed)	Growth rate of R&D investment
	B3	Science and Technology Policy Scenario (High-speed)	
	A4	Healthcare Policy Scenario (Low-speed)	Rate of increase in the number of beds and rate of increase in the number of doctors
	B4	Healthcare Policy Scenario (High-speed)	
	A5	Education Policy Scenario (Low-speed)	Growth rate of number of schools, growth rate of teachers and growth rate of the number of students in school
	B5	Education Policy Scenario (High-speed)	
Policy mix scenarios	C	Low-speed development scenario	–
	D	High-speed development scenario	–

We divided the forecast years into five phases, namely 2020–2025, 2026–2030, 2031–2035, 2036–2040, and 2041–2050, and adjusted the parameters in the different scenarios according to these five phases. Specific parameter settings are given in Table 7. To make the table more concise, ‘O’ is used for benchmark scenarios, ‘L’ for low-speed scenarios and ‘H’ for high-speed scenarios.

**Table 7:** Scenario’s parameter setting.

Indicator			2020–2025	2026–2030	2031–2035	2036–2040	2041–2050
Birth Policy Scenario	Natural population growth rate	O	0.00770				
		L	0.00696				
		H	0.00850				
Employment Policy Scenario	Average wage growth rate	O	0.15840	0.14170	0.13480	0.12170	0.12930
		L	0.14256	0.12753	0.12132	0.10953	0.11637
		H	0.17424	0.15587	0.14828	0.13387	0.14223
	Coefficient of employment	O	0.02000				
		L	0.01800				
		H	0.02200				
Science and Technology Policy Scenarios	Growth rate of R&D investment	O	0.15710	0.12860	0.11960	0.10260	0.09560
		L	0.01571	0.01286	0.01196	0.01026	0.00956
		H	0.39275	0.32150	0.29900	0.25650	0.23900
Healthcare Policy scenarios	Rate of increase in the number of beds	O	0.07870	0.07230	0.06590	0.06190	0.07840
		L	0.00787	0.00723	0.00659	0.00619	0.00784
		H	0.19675	0.18075	0.16475	0.15475	0.19600
	Rate of increase in the number of doctors	O	0.09590	0.08290	0.06980	0.05450	0.06910
		L	0.00959	0.00829	0.00698	0.00545	0.00691
		H	0.23975	0.20725	0.17450	0.13625	0.17275
Education Policy Scenarios	Growth rate of number of schools	O	0.01530	0.01300	− 0.00660	− 0.01160	− 0.01660
		L	0.00153	0.00130	− 0.00726	− 0.01740	− 0.02822
		H	0.03825	0.03250	− 0.00462	− 0.00812	− 0.01162
	Growth rate of teachers	O	0.03950	0.02650	0.01940	0.01810	0.03220
		L	0.00395	0.00265	0.00194	0.00181	0.00322
		H	0.09875	0.06625	0.04850	0.04525	0.08050
	Growth rate of the number of students in school	O	0.05070	0.04010	0.01940	− 0.00060	0.01560
		L	0.00507	0.00401	0.00194	− 0.00010	0.00256
		H	0.12675	0.10025	0.04850	0.00050	0.03900

The guidelines used to set parameter values for each variable within a given scenario are as follows.

1. From the relationship between the parameters of each scenario, an increase in the natural rate of population growth will promote the development of employment, science and technology, healthcare and education, which in turn will promote population growth. Moreover, employment, technology, healthcare, and education mutually reinforce each other. Consequently, we posit that all parameters of the five policies have positive effects and assign the parameter values accordingly.
2. From the relationship between parameters of different scenarios, the parameter values of each variable in the baseline scenario are reduced by 10% and increased by 10% as the parameter settings in the low- and high-speed scenarios, respectively. Through many experiments, it is found that the influence of science and technology policy factors, medical policy factors and education policy factors on population development situation is weak, significantly lower than that of fertility policy factors and employment policy factors. In order to more significantly reflect the effect difference of different policies in the simulation results, the parameter control intensity of science and technology policy, medical policy and education policy scenarios is changed, that is, the parameter values of each variable in the baseline scenario are reduced by 90% and increased by 150% as the parameter Settings in the low-speed scenario and high-speed scenario, respectively. Among them, variables with negative parameter values are adjusted again in several experiments. In summary, we believe that the amplitude of variation of each regulatory parameter is the same in the baseline scenario, the low-speed scenario and the high-speed scenario, and that it is feasible to study the extent to which the five policies affect the changes in population development.

### Simulation result analysis

Based on the scenario parameters set in the above section, we simulate the future population situation in Xi’an and obtain the trend map of population change under different scenarios. Supplementary Table S1 shows the simulation results of Xi’an population.

**Figure 8:**
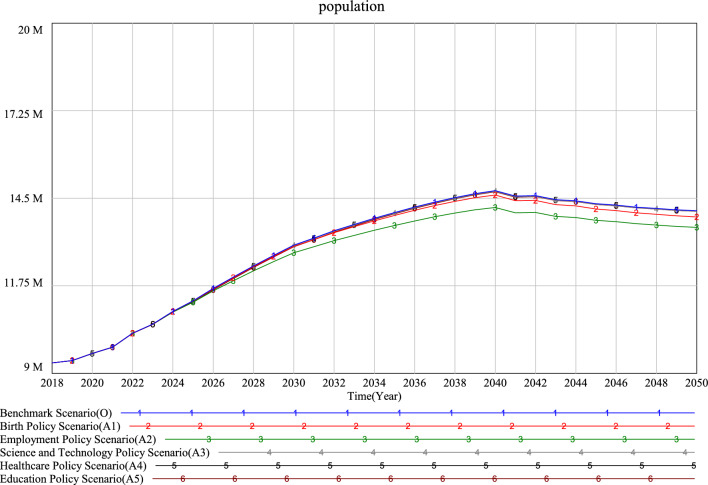
Simulation results of the Xi’an population under different single low speed scenarios.

**Figure 9:**
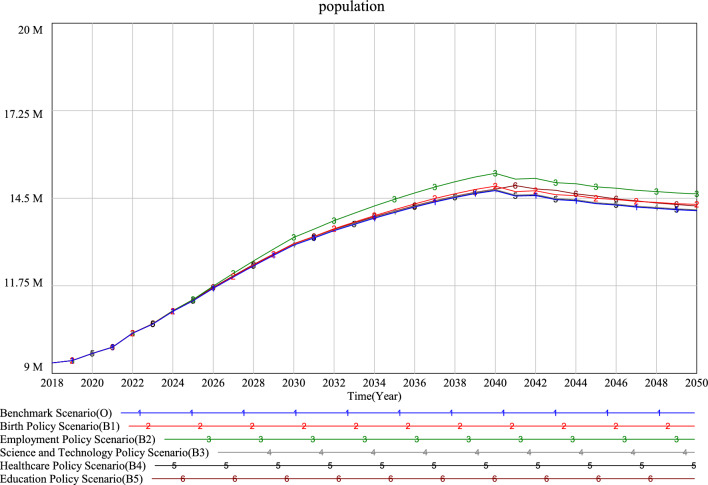
Simulation results of the Xi’an population under different single high speed scenarios.

**Figure 10:**
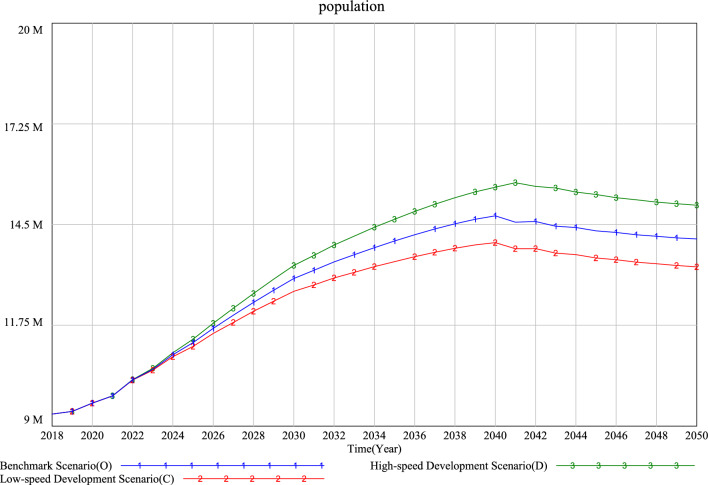
Simulation results of the Xi’an population under different policy mix scenarios.

According to the simulation results (Figs. 8, 9 and 10), it can be seen that the changing trend of the future population in all scenarios will rise first and then fall. The population prediction results of each scenario at the simulation deadline (2050) are greater than those of the initial year (2019), which indicates that the future population of each scenario will increase compared with 2019.

1. Analysis of single policy scenarios simulation results.

Figure 8 shows that the population in the benchmark scenario gradually increases from 9,398,388 in 2019 to 14,739,242 in 2040, and then gradually decreases to 14,107,820 in 2050. The birth, employment, science and technology, healthcare, and education policy scenarios also peaked in 2040 under the low speed mode (scenarios A1, A2, A3, A4, and A5), with their peaks being 14,600,846, 14,210,083, 14,718,157, 14,728,837 and 14,700,912 respectively. By 2050, the predicted population for these five scenarios will slowly decline to 13,911,854, 13,583,951, 14,088,781, 14,100,696, and 14,087,582, respectively, which is 195,966, 523,869, 19,039, 7124, and 2,0238 fewer than the population prediction predicted by the baseline scenario. From this result, it can be found that when the government policy support for birth, employment, science and technology, healthcare, and education is weakened, the population growth trend from 2019 to 2050 will decrease overall. In addition, the impact of the five policies on future population development is in the following order: employment policy  >   birth policy  > education policy  > science and technology policy > healthcare policy. Of these, the employment policy and the birth policy have considerably more significant effects on population development than the remaining three policies.

According to Fig. 9, different from the previous low speed mode, the time for Xi’an to reach the population peak under the high speed mode of five policy scenarios (scenarios B1, B2, B3, B4, and B5) is slightly different. Among them, birth (scenario B1), employment (scenario B2), technology (B3), and healthcare policy (scenario B4) peak in 2040, while education policy (scenario B5) reaches the population peak in 2041. According to the population forecast results of various policy scenarios in 2040, the ranking of the impact of each policy scenario on population development from strong to weak can be obtained: Employment policy scenario (scenario B2) still ranks first, with a peak population of 15,281,558. Birth policy scenario (scenario B1) ranked second, with a peak population of 14,878,788. Education policy scenario (scenario B5) was 14,787,770. 14,774,415 for the science and technology policy scenario (scenario B3) and 14,750,786 for the healthcare policy scenario (scenario B4). ​This trend can be seen in the results: employment policy, birth policy, education policy, science and technology policy, and healthcare policy have a gradually weaker impact on population development, while employment policy and birth policy have a more significant effect. ​This result can also be found in the population simulation results for 2050. The predicted population of Xi’an in 2050 under the employment policy and the birth policy in the high-speed mode is 146,636,910 and 145,306,256, respectively, which is 529,090 and 198,436 higher than the baseline scenario. The population outcomes predicted by the healthcare policy in the high-speed mode are only 9,408 more than in the benchmark scenario.

Based on the above analysis, the following conclusions can be drawn.

Conclusion 1: In the single policy scenario, the influence of employment policy, birth policy, education policy, science, and technology policy and healthcare policy on the future population development gradually decreases.

Conclusion 2: The impact of the employment policy and the birth policy on the future population development is significantly more significant than the impact of the other three policies. This phenomenon is pronounced in the low speed mode of the five single policy scenarios. Therefore, the government should pay attention to the support of employment and birth policies when formulating the population development plans. First of all, the purpose of strengthening employment policy support is to attract non-local people to move in, and solve the problems of difficult employment and low income by raising the level of working income or setting up more jobs, which has an essential impact on the choice of residence for non-local people. It can be seen that employment policies play a crucial role in attracting population migration, which explains why employment policies are ranked first in the simulation results. Second, increasing support for birth policies is one of the most direct measures for population improvement, affecting current population growth and future population growth after these newcomers enter marriage and childbearing. Therefore, the birth policy also has a significant impact on the future population. We believe that the effect of the birth policy is lower than that of the employment policy, which can be explained by “starting a job before starting a family”. Without the material foundation laid by “starting a job”, how can we reproduce the next generation after “starting a family”? Thus, in the single policy scenario, the effect of employment policy on population development is more significant than that of birth policy.

Conclusion 3: Compared with employment policy and birth policy, although education, science and technology, and healthcare policy also have a specific impact on population development, the impact intensity is very different, especially the healthcare policy. The emergence of this result because of the education, science and technology, and healthcare approach in promoting the living standard of the residents have a significant effect, such as education policy can provide an enabling environment for training the next generation, science and technology policy can help more convenient life, healthcare policy can bring security to the health of the residents, but they are on the move attractive compared with employment policies have apparent difference.

2. Analysis of policy mix scenarios simulation results.

Figure 10 shows an obvious trend: The population simulation results of the low-speed development scenario (scenario C) are significantly smaller than those of the benchmark scenario (scenario O) and the population simulation results of the high-speed development scenario (scenario D) are significantly larger than those of the benchmark scenario (scenario O). The peak population of the low-speed development scenario (scenario C) is 14,005,866 in 2040, the peak population of the benchmark scenario (scenario O) is 14,739,242 in 2040, and the peak population of the high-speed development scenario (scenario D) is 15,219,496 in 2042. In 2050, the population of low-speed development scenario (scenario C), benchmark scenario (scenario O) and high-speed development scenario (scenario D) will be 13,426,816, 14,107,820 and 14,802,612, respectively. That is, the low-speed development scenario (scenario C) has 681,004 fewer people than the benchmark scenario (scenario O), and the high-speed development scenario (scenario D) has 694,792 more people than the benchmark scenario (scenario O). Compared with the single policy scenarios, the combined policy scenarios has a higher significant impact on population development, which indicating that the coordinated implementation of multiple policies will bring greater help in improving the future population.

3. Summary of analysis.

According to the above analysis results, both single policy scenarios and combination policy scenarios have an impact on the population development and the order of impact intensity from large to minor is: portfolio policy > employment policy > birth policy > education policy > science and technology policy > medical policy. Under all scenarios, the population of Xi’an will reach its peak around 2040. In the portfolio policy, the peak population of the high-speed development scenario is 694,792 more than the peak population of the benchmark scenario. The impact on population development is significantly more significant than in the high-speed model with five single policy scenarios. This is because the government has also strengthened the support for birth, employment, science and technology, healthcare and education policies and paid for more personnel, funds and resources. Therefore, the combined policy is the optimal choice to improve the future population. Nonetheless, it also requires the government to invest further resources, which may lead to unavoidable economic development pressures and environmental energy problems, and is not conducive to the city’s overall development goals. Hence, which policy to adopt depends on the government’s ability to implement the policy and allocate resources.

In summary, population development planning requires comprehensive consideration of five policies. As the best option, the combination policy needs to be fully considered based on an assessment of the existing environment, personnel, economy and long-term economic development planning. Reducing some policy support is also a sensible option if the government cannot ensure sufficient capacity or resources to support the coordinated implementation of the five policies. Our proposal is this: since employment and birth policies have a significant impact on population development, we propose to strengthen support for employment and birth policies, with the option to appropriately reduce investment in education, science and technology, or health care policies. Of course, to realize the healthy growth of the urban population and achieve the overall goal of social development, the government should actively adjust the population development plan according to the changing economic and social development situation.

## Conclusions

Population is closely related to economic development, social stability, employment of the workforce, and sustainable utilization of resources. Having a scientific population development plan has an essential impact on the achievement of city’s long-term economic development goals. To accurately reflect the urban population change trend and provide help for the government to formulate a scientific and practical population development plan, we used system dynamics to model the urban population prediction system. To explore the impact of different policies on the future development trends of the population, we take Xi’an City as an example to conduct the study. That is, based on birth, employment, science and technology, healthcare, and education policies, we set benchmark scenarios, single policy scenarios and policy mix scenarios, and dynamically simulated the demographic development trend in Xi’an from 2019 to 2050.

In addition, to address the issue of intense subjectivity in setting the parameters of the system dynamics model, we propose a multi-objective lioness optimization algorithm based on the single-objective lioness optimization algorithm, and use this algorithm to automatically optimize some critical parameters in the system dynamics model. The introduction of the multi-objective lioness optimization algorithm enables accurate simulation of historical data by predictive models. It provides a guarantee for the accurate prediction of the future population. The main conclusions of this paper are as follows.

1. By analyzing numerous factors to determine the relevant indexes of the population system, a dynamic model of urban population prediction system is constructed, and some critical parameters of the model are optimized by the multi-objective lioness optimization algorithm. Taking Xi'an City as an example, the simulation analysis is carried out. On historical inspection, the model performed well and was able to fit the historical demographic development trends of Xi’an well. Therefore, it is feasible to use predictive models to model the future population of Xi'an city. Due to the similarity in the influence factors and development trends of population development, we believe that our model is also suitable for future population projections in other cities.
2. If the population of Xi’an keeps the current development trend, the people of Xi’an will continue to rise in the next few years until it reaches the peak of 14,739,242 in 2040, which is 1.57 times the population of Xi’an in 2019.
3. In general, the positive influence of employment policy, birth policy, education policy, science and technology policy, and healthcare policy on the population development has gradually weakened. Among the five policies, the employment policy and the birth policy have a significantly more significant impact on population development than the other three policies. Therefore, strengthening support for employment policies is the first way to promote population improvement. Although the effect of the birth policy is weaker than that of the employment policy, it also has an essential influence on population development.

In addition, the urban population prediction model based on multi-objective lioness optimization algorithm and system dynamics shows superior accuracy in historical data simulation, but it still suffers from some drawbacks. First, the urban population prediction system is a dynamic system involving various influences. It is difficult to consider all the influencing features in the model design. We have chosen only a few representative factors in the study and have deliberately simplified the existing population system. Thus, there is some subjectivity in the choice of indices and the design of index relations, which leads to a partial deviation of our constructed system dynamics model from the existing population evolution system. Second, how to further optimize the urban population prediction model by combining it with other methods or theories remains to be further investigated. Finally, policies involved in the population development plan are being expanded, such as improving public services, guaranteeing elderly and child care services, optimizing transportation, improving air quality and strengthening the ecological environment. The implementation of these policies will inevitably have an impact on population development. How to incorporate these policies into the population prediction model and quantify their impact on the future population development of cities, will be our next line of work.

## Supplementary Information

Supplementary Table S1.

## Data Availability

The datasets generated during the current study are available from the corresponding author on reasonable request.
