# Supplementary material for: Urban population prediction based on multi-objective lioness optimization algorithm and system dynamics model
Source: Sci Rep. 2023 Jul 22;13:11836. doi: 10.1038/s41598-023-39053-1 (PMC10363121; doi:10.1038/s41598-023-39053-1)
Supplement: Supplementary file 1 — Supplementary Table S1. [file 41598_2023_39053_MOESM1_ESM.docx]

| **Supplementary Table S1**. Simulation results of Xi’an population. | | | | | | | | | | | | | |
| --- | --- | --- | --- | --- | --- | --- | --- | --- | --- | --- | --- | --- | --- |
| Time (Year) | Benchmark scenario | Single policy scenario (Low speed) | | | | | Single policy scenario (High speed) | | | | | Policy mix scenario | |
|  |  | Education Policy Scenario | Healthcare Policy Scenario | Science and Technology Policy Scenario | Employment Policy Scenario | Birth Policy Scenario | Education Policy Scenario | Healthcare Policy Scenario | Science and Technology Policy Scenario | Employment Policy Scenario | Birth Policy Scenario | Low speed development scenario | High-speed development scenario |
| 2000 | 7411400 | 7411400 | 7411400 | 7411400 | 7411400 | 7411400 | 7411400 | 7411400 | 7411400 | 7411400 | 7411400 | 7411400 | 7411400 |
| 2001 | 7361200 | 7361200 | 7361200 | 7361200 | 7361200 | 7361200 | 7361200 | 7361200 | 7361200 | 7361200 | 7361200 | 7361200 | 7361200 |
| 2002 | 7726778 | 7726778 | 7726778 | 7726778 | 7726778 | 7726778 | 7726778 | 7726778 | 7726778 | 7726778 | 7726778 | 7726778 | 7726778 |
| 2003 | 7856078 | 7856078 | 7856078 | 7856078 | 7856078 | 7856078 | 7856078 | 7856078 | 7856078 | 7856078 | 7856078 | 7856078 | 7856078 |
| 2004 | 8061564 | 8061564 | 8061564 | 8061564 | 8061564 | 8061564 | 8061564 | 8061564 | 8061564 | 8061564 | 8061564 | 8061564 | 8061564 |
| 2005 | 8084500 | 8084500 | 8084500 | 8084500 | 8084500 | 8084500 | 8084500 | 8084500 | 8084500 | 8084500 | 8084500 | 8084500 | 8084500 |
| 2006 | 8313988 | 8313988 | 8313988 | 8313988 | 8313988 | 8313988 | 8313988 | 8313988 | 8313988 | 8313988 | 8313988 | 8313988 | 8313988 |
| 2007 | 8457497 | 8457497 | 8457497 | 8457497 | 8457497 | 8457497 | 8457497 | 8457497 | 8457497 | 8457497 | 8457497 | 8457497 | 8457497 |
| 2008 | 8683210 | 8683210 | 8683210 | 8683210 | 8683210 | 8683210 | 8683210 | 8683210 | 8683210 | 8683210 | 8683210 | 8683210 | 8683210 |
| 2009 | 8930853 | 8930853 | 8930853 | 8930853 | 8930853 | 8930853 | 8930853 | 8930853 | 8930853 | 8930853 | 8930853 | 8930853 | 8930853 |
| 2010 | 9162339 | 9162339 | 9162339 | 9162339 | 9162339 | 9162339 | 9162339 | 9162339 | 9162339 | 9162339 | 9162339 | 9162339 | 9162339 |
| 2011 | 9267081 | 9267081 | 9267081 | 9267081 | 9267081 | 9267081 | 9267081 | 9267081 | 9267081 | 9267081 | 9267081 | 9267081 | 9267081 |
| 2012 | 9233158 | 9233158 | 9233158 | 9233158 | 9233158 | 9233158 | 9233158 | 9233158 | 9233158 | 9233158 | 9233158 | 9233158 | 9233158 |
| 2013 | 9195237 | 9195237 | 9195237 | 9195237 | 9195237 | 9195237 | 9195237 | 9195237 | 9195237 | 9195237 | 9195237 | 9195237 | 9195237 |
| 2014 | 9067657 | 9067657 | 9067657 | 9067657 | 9067657 | 9067657 | 9067657 | 9067657 | 9067657 | 9067657 | 9067657 | 9067657 | 9067657 |
| 2015 | 9233207 | 9233207 | 9233207 | 9233207 | 9233207 | 9233207 | 9233207 | 9233207 | 9233207 | 9233207 | 9233207 | 9233207 | 9233207 |
| 2016 | 9212296 | 9212296 | 9212296 | 9212296 | 9212296 | 9212296 | 9212296 | 9212296 | 9212296 | 9212296 | 9212296 | 9212296 | 9212296 |
| 2017 | 9423838 | 9423838 | 9423838 | 9423838 | 9423838 | 9423838 | 9423838 | 9423838 | 9423838 | 9423838 | 9423838 | 9423838 | 9423838 |
| 2018 | 9326076 | 9326076 | 9326076 | 9326076 | 9326076 | 9326076 | 9326076 | 9326076 | 9326076 | 9326076 | 9326076 | 9326076 | 9326076 |
| 2019 | 9398388 | 9398388 | 9398388 | 9398388 | 9398388 | 9398388 | 9398388 | 9398388 | 9398388 | 9398388 | 9398388 | 9398388 | 9398388 |
| 2020 | 9624028 | 9623905 | 9623419 | 9624028 | 9624028 | 9624028 | 9624233 | 9625042 | 9624028 | 9624028 | 9624028 | 9623296 | 9625248 |
| 2021 | 9821874 | 9821631 | 9820652 | 9821874 | 9821873 | 9821874 | 9822207 | 9823572 | 9821874 | 9821874 | 9821874 | 9820409 | 9823906 |
| 2022 | 10258911 | 10256674 | 10256663 | 10258613 | 10258908 | 10258152 | 10262433 | 10261706 | 10259408 | 10258914 | 10259670 | 10253368 | 10266487 |
| 2023 | 10546148 | 10537325 | 10542662 | 10545321 | 10538802 | 10543990 | 10559026 | 10549870 | 10547528 | 10553496 | 10548308 | 10523517 | 10573638 |
| 2024 | 10945403 | 10925798 | 10940007 | 10943676 | 10924304 | 10940904 | 10967287 | 10950328 | 10948284 | 10966502 | 10949905 | 10893122 | 11000727 |
| 2025 | 11277156 | 11234287 | 11268725 | 11274211 | 11233835 | 11269408 | 11310547 | 11283251 | 11282068 | 11320545 | 11284910 | 11172038 | 11372835 |
| 2026 | 11666236 | 11625110 | 11657676 | 11661661 | 11591691 | 11654167 | 11701458 | 11673011 | 11673865 | 11741036 | 11678315 | 11525702 | 11803062 |
| 2027 | 12022960 | 11973387 | 12013181 | 12016797 | 11908210 | 12005490 | 12063211 | 12030526 | 12033237 | 12138413 | 12040452 | 11825996 | 12214654 |
| 2028 | 12374702 | 12326791 | 12364866 | 12366854 | 12219670 | 12350683 | 12416135 | 12382864 | 12387791 | 12531105 | 12398760 | 12131060 | 12618828 |
| 2029 | 12704633 | 12652956 | 12694147 | 12695186 | 12507366 | 12672898 | 12748996 | 12713450 | 12720390 | 12904238 | 12736436 | 12405539 | 13006434 |
| 2030 | 13026696 | 12975752 | 13016076 | 13015657 | 12788322 | 12986036 | 13072227 | 13036070 | 13045109 | 13268508 | 13067464 | 12676936 | 13384527 |
| 2031 | 13249612 | 13196501 | 13238775 | 13237274 | 12974483 | 13199264 | 13296284 | 13259389 | 13270194 | 13529310 | 13300127 | 12850131 | 13659297 |
| 2032 | 13480731 | 13434914 | 13469874 | 13467068 | 13169718 | 13420532 | 13527858 | 13490887 | 13503524 | 13797502 | 13541164 | 13041604 | 13940956 |
| 2033 | 13676892 | 13631177 | 13665962 | 13662056 | 13332688 | 13606664 | 13724668 | 13687355 | 13701645 | 14028010 | 13747438 | 13193716 | 14185010 |
| 2034 | 13870306 | 13832156 | 13859400 | 13854312 | 13493888 | 13790085 | 13921048 | 13881048 | 13896989 | 14254798 | 13950936 | 13351348 | 14427648 |
| 2035 | 14050268 | 14009210 | 14039364 | 14033217 | 13643700 | 13960024 | 14094144 | 14061242 | 14078716 | 14465996 | 14141025 | 13487490 | 14644356 |
| 2036 | 14221786 | 14184517 | 14210941 | 14203724 | 13786400 | 14121612 | 14267929 | 14232960 | 14251920 | 14667376 | 14322586 | 13621040 | 14860401 |
| 2037 | 14379494 | 14341304 | 14368712 | 14360532 | 13917242 | 14269466 | 14422658 | 14390826 | 14411132 | 14852887 | 14490270 | 13737050 | 15054718 |
| 2038 | 14520870 | 14484820 | 14510190 | 14501090 | 14033886 | 14401162 | 14565064 | 14532316 | 14553868 | 15019815 | 14641454 | 13835576 | 15234158 |
| 2039 | 14645070 | 14608678 | 14634509 | 14624582 | 14135640 | 14515854 | 14688382 | 14656591 | 14679250 | 15167136 | 14775296 | 13850613 | 15391174 |
| 2040 | 14739242 | 14700912 | 14728837 | 14718157 | 14210083 | 14600846 | 14787770 | 14750786 | 14774415 | 15281558 | 14878788 | 14005866 | 15519198 |
| 2041 | 14566214 | 14532576 | 14556412 | 14545546 | 14041880 | 14422756 | 14897246 | 14577282 | 14600687 | 15102432 | 14710914 | 13857342 | 15131386 |
| 2042 | 14583344 | 14550707 | 14573874 | 14562624 | 14053460 | 14432760 | 14794035 | 14594224 | 14617906 | 15124595 | 14735292 | 13828799 | 15219496 |
| 2043 | 14454800 | 14425428 | 14445833 | 14434483 | 13930850 | 14299166 | 14748947 | 14465291 | 14488682 | 14988726 | 14611900 | 13689744 | 15025852 |
| 2044 | 14420612 | 14392572 | 14411972 | 14400408 | 13895035 | 14258772 | 14636212 | 14430898 | 14454302 | 14955505 | 14584041 | 13606288 | 15044792 |
| 2045 | 14328100 | 14302286 | 14319841 | 14308208 | 13806230 | 14160976 | 14567922 | 14338115 | 14361268 | 14858170 | 14496934 | 13688620 | 14934654 |
| 2046 | 14284686 | 14260094 | 14276701 | 14264943 | 13761914 | 14111649 | 14478754 | 14294540 | 14317604 | 14815064 | 14459571 | 13592587 | 14926369 |
| 2047 | 14220625 | 14197571 | 14212920 | 14201116 | 13699388 | 14042068 | 14418032 | 14230304 | 14253150 | 14748626 | 14401168 | 13586706 | 14862824 |
| 2048 | 14181398 | 14159340 | 14173908 | 14162038 | 13659092 | 13997000 | 14351284 | 14190968 | 14213672 | 14709966 | 14367936 | 13509754 | 14848400 |
| 2049 | 14137878 | 14116888 | 14130592 | 14118702 | 13615519 | 13947782 | 14303568 | 14147346 | 14169847 | 14665872 | 14330274 | 13482360 | 14813128 |
| 2050 | 14107820 | 14087582 | 14100696 | 14088781 | 13583951 | 13911854 | 14255773 | 14117228 | 14139556 | 14636910 | 14306256 | 13426816 | 14802612 |
